# Piezoelectric-AlN resonators at two-dimensional flexural modes for the density and viscosity decoupled determination of liquids

**DOI:** 10.1038/s41378-022-00368-0

**Published:** 2022-04-02

**Authors:** Linya Huang, Wei Li, Guoxi Luo, Dejiang Lu, Libo Zhao, Ping Yang, Xiaozhang Wang, Jiuhong Wang, Qijing Lin, Zhuangde Jiang

**Affiliations:** 1 State Key Laboratory for Manufacturing Systems Engineering, International Joint Laboratory for Micro/Nano Manufacturing and Measurement Technologies, Overseas Expertise Introduction Center for Micro/Nano Manufacturing and Nano Measurement Technologies Discipline Innovation, Xi’an Jiaotong University (Yantai) Research Institute for Intelligent Sensing Technology and System, Xi’an Jiaotong University, Xi’an, 710049 China; 2 School of Mechanical Engineering, Xi’an Jiaotong University, Xi’an, 710049 China; 3 The Eleventh Research Institute of The Sixth Academy of CASC, Xi’an, 710100 China

**Keywords:** Electrical and electronic engineering, Chemistry

## Abstract

A micromachined resonator immersed in liquid provides valuable resonance parameters for determining the fluidic parameters. However, the liquid operating environment poses a challenge to maintaining a fine sensing performance, particularly through electrical characterization. This paper presents a piezoelectric micromachined cantilever with a stepped shape for liquid monitoring purposes. Multiple modes of the proposed cantilever are available with full electrical characterization for realizing self-actuated and self-sensing capabilities. The focus is on higher flexural resonances, which nonconventionally feature two-dimensional vibration modes. Modal analyses are conducted for the developed cantilever under flexural vibrations at different orders. Modeling explains not only the basic length-dominant mode but also higher modes that simultaneously depend on the length and width of the cantilever. This study determines that the analytical predictions for resonant frequency in liquid media exhibit good agreement with the experimental results. Furthermore, the experiments on cantilever resonators are performed in various test liquids, demonstrating that higher-order flexural modes allow for the decoupled measurements of density and viscosity. The measurement differences achieve 0.39% in density and 3.50% in viscosity, and the frequency instability is below 0.05‰. On the basis of these results, design guidelines for piezoelectric higher-mode resonators are proposed for liquid sensing.

## Introduction

Monitoring the properties of a liquid has provided an important platform for resonant devices based on micro- and nanoelectromechanical system (MEMS and NEMS) technology to achieve miniaturization and portability^1,2^. Among these properties, density and viscosity are regarded as key quantities of a liquid in various industries, such as the process monitoring of (bio)chemical reactions^3,4^, the weight evaluation of active particles^5,6^, and the concentration control of solutions for extraction^7^. In general, dynamic mode cantilevers have received extensive attention in physical and chemical sensing. The fundamental out-of-plane bending is a typical vibrating mode of cantilevers that can be flexibly excited by electrostatic^8^, piezoelectric^9,10^, photothermal^11^, and magnetic^12,13^ forces. Meanwhile, torsional vibration^14–16^ is used as an alternative for enhancing the sensing behavior of cantilevers. These conventional vibrations of a cantilever-based resonator have been shown to present two separate dependencies of resonant frequency on density and Q-factor on viscosity.

Cantilever-based resonators under liquid immersion have consistently proposed challenges for overcoming high viscous damping. This condition requires resonators to raise vibration orders or excite nonconventional vibrations because the hydrodynamic force^17,18^ can be influenced by the vibrational mode, which then manages resonance behavior. For example, the in-plane mode^19,20^ has been adopted to increase the Q-factor of a resonator by transferring the shear force rather than the compressive force to the fluid. A higher vibration mode^21–23^ enhances the Q-factor by decreasing the vibration amplitude of cantilevers owing to a higher modal stiffness. However, an inherent problem for these vibrations is that resonant magnitude is a function of the product of the liquid density and viscosity^24^. This condition restricts a decoupled solution for these vibrations. Furthermore, the pure shear forces generated by a resonator operating in in-plane modes, such as in-plane bending and extensional modes, make determining density and viscosity independently impossible. This scenario can be described by the second Stokes problem^24,25^. With regard to measurement performance, Toledo et al.^26^ presented a piezoelectric microresonator resonating at fourth-order vibrations, which addressed the density and viscosity by using four calibrated coefficients. The mean deviations are 0.38% for density (0.98–1.08 g/ml) and 7.36% for viscosity (1.71–1.97 cP). Bircher et al.^27^ presented a nanomechanical resonator vibrating at the third mode. For the single calibrated coefficient, the mean deviations are 3.2% for density (998–1154 kg m^−3^) and 10.1% for viscosity (1–10.5 cP). While using three calibrations, the mean deviations are 0.8% for density and 3.2% for viscosity. By comparison, a commercial density–viscosity meter (Anton Paar 4101, Lovis 2000) reaches 0.03% for density (0–3 g/ml) and 0.5% for viscosity (0.3–10000 cP). Thus, the performance of MEMS resonant sensors remains a critical concern for liquid sensing, although this technology has been regarded as an essential solution due to its real-time and portable operation.

When focusing on the output interface of resonators, optical characterization is still a common approach^14,27,28^; it detects slight deflections even in the sub-Angstrom regime, but it is bulky and alignment-dependent. Thin-film piezoelectric-on-silicon technology offers full electrical interfaces, including input and output transductions, for resonators to simplify their design by applying piezoelectric self-actuation and self-sensing methods. However, only a few studies have taken full advantage of this capability because resonance amplitude decreases dramatically in liquid media, making maintaining electrical access a challenge, particularly for microscale or nanoscale resonators vibrating in higher-order modes.

Here, we present a wide-stepped microcantilever resonator for piezoelectrically actuating higher-order nonconventional vibrations. The resonator aims to simultaneously reduce viscous losses and circumvent the limited product function with the density and viscosity to establish a separate function for them by resonant parameters, which is used as a solution to enhance the performance of the density and viscosity decoupling determination. Higher-order vibrations feature moderate frequencies within the kHz range, allowing self-sensing by reading out the piezoelectrically induced voltage. To the best of our knowledge, these two-dimension modal dynamics based on a plate-structural cantilever have not been studied in detail, although a cantilever naturally satisfies the inviscid fluid condition and has been widely employed for liquid monitoring. This study analytically modeled the two-dimensional flexural modes to characterize the modal width effect of the plate cantilever, verifying it from theoretical and experimental results. Experiments were performed to determine the density and viscosity of the measured liquid by using separate estimation equations with only a single calibration. Finally, output characteristics were discussed and design methods were presented, particularly for piezoelectric cantilevers in higher-order flexural modes in liquid media.

## Materials and methods

### Resonator design and fabrication

**Fig. 1:**
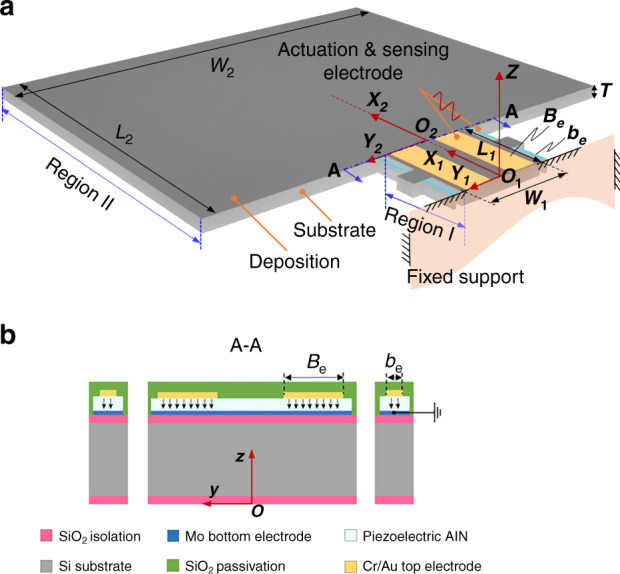
Overall design of the stepped piezo-cantilever resonator. **a** Detailed components of the resonator under the spatial coordinate system. **b** A-A cross-sectional schematic of the resonator, including the directional arrows to indicate the electrical field.

The micromachined cantilever is designed with a stepped shape, as shown in Fig. 1a. It consists of a support beam (Region I) and a cantilever plate (Region II). The actuation and sensing electrodes are separated by different beams. Two of the electrodes with an active piezoelectric layer width of *B*_e_ are used for actuation, while the other electrodes with an piezoelectric layer width of *b*_*e*_ are used for sensing. The electrodes with width *b*_*e*_ are fabricated on four slender sensitive beams (length × width = 129 μm × 18 μm) for concentrating on the deformation strain. The support beam is designed with a length of *L*_1_ = 318 μm and a width of *W*_1_ = 246 μm. Meanwhile, the cantilever plate has a length of *L*_2_ = 1089 μm and a width of *W*_2_ = 1382 μm. The thickness *T* of the micromachined cantilever is 25 μm. Both *B*_*e*_ and *b*_*e*_ represent the widths of the top electrodes, which should be enlarged to enhance the self-actuation and self-sensing capabilities of the piezo-cantilever resonator. In addition, to compromise the tolerance width in the fabrication process, Be and be are designed with the corresponding values of 80 μm and 10 μm, respectively. The proposed cantilever with a plate structure and a wide step-change along the width is designed to generate the width effect. Thus, the out-of-plane mode is determined by the vibrations not only along the length but also along the width. In this manner, the two-dimensional mode can be nonconventionally excited with respect to the slender cantilever. The smaller width of Region I is used to adjust the higher-order modal stiffness with a moderate value to enhance resonance stability.

**Fig. 2:**
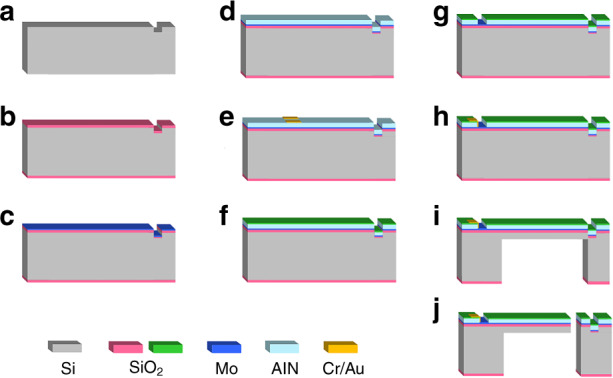
Fabrication process of the stepped piezo-cantilever resonator. **a** Making marks by RIE; **b** thermal oxidization process; **c** Mo layer sputtering; **d** RMS AlN deposition; **e** top Cr/Au electrode sputtering; **f** LPCVD SiO2 passivation; **g** via dry etching to expose the bottom electrode; **h** the wire fabrication by lift-off process; **i** backside DRIE; and **j** DRIE releasing the cantilever.

The piezo-cantilever resonators are fabricated in 4-inch Si wafers, and the cross-sectional schematic is displayed in Fig. 1b. Both sides of the wafer are covered by 400 nm thermal silicon oxide layers as electrical isolations. Then, a 130 nm Mo layer is deposited by sputtering technology onto the silicon oxide layer as the bottom electrode of the cantilever. The Mo electrode without patterning contributes to increasing availability for flexural modes by guaranteeing the in-phase sinusoidal drive applied to a parallel connection of the electrode pairs. The piezoelectric aluminum-nitride (AlN) film with a thickness of 1 μm was deposited by reactive magnetron sputtering (RMS) technology. Then, the top electrodes consisting of chromium and gold films are fabricated with thicknesses of 20 nm and 200 nm, respectively. Thereafter, the wafer is covered with a SiO_2_ layer via low-pressure chemical vapor deposition (LPCVD). This layer is used as a passivation layer for the resonator. Finally, the stepped piezo-cantilever is released by etching technology to obtain the proposed resonator. The fabrication process flow is shown in Fig. 2.

### Modal analyses

The resonant frequency and mode shape in vacuum represent natural features of a piezo-cantilever, which are the foundation for expressing resonance behavior in liquid media. For the proposed piezo-cantilever resonator, Regions I and II of the cantilever play a dominant role in its resonance behavior, as shown in Fig. 1a. Meanwhile, the four slender sensitive beams have a considerably smaller volume, and thus they can be reasonably disregarded in the subsequent analyses. In addition, classic plate theory is adopted because the thickness is considerably less than length and width (including Regions I and II). The modeling analyses of the cantilever are based on the following assumptions^29,30^. (1) The material is linearly elastic and transversely isotropic; (2) the out-of-plane vibration mode is dominant and its deformation is small, so the shear and nonlinear deformation are neglected; (3) the piezoelectric strain coefficient *d*_31_ (or *d*_32_) is constant; and (4) the transverse normals remain perpendicular to the middle surface after deformation. The theoretical modeling is analyzed in the Supplementary Information, and Table 1 lists the main resonance parameters of the cantilever under the first six orders of flexural vibrations. The first six eigenvalues of *Λ*_*i*_ = *β*_*i*_*L*, in which *L* is the whole length of the cantilever, have notable changes compared with the conventional values, where *Λ*_1_ = 1.8751, *Λ*_2_ = 4.6941, and *Λ*_3_ = 7.8548^17,31^. This indicates that the stepped cantilever has the capability to excite distinctive mode shapes in both vacuum and liquid-phase environments.

**Table 1:** Resonant parameters of the cantilever by theoretical results

Order	*Λ*_*i*_	*f*_vac_ (kHz)
1st	1.3240	7.9636
2nd	4.2702	82.8409
3rd	7.3389	234.8950
4th	10.6355	493.3185
5th	14.1685	875.5030
6th	17.6156	1353.3413

The preceding analyses require a more detailed correction in accordance with certain mode shapes for two reasons. (i) Considering that the wide-stepped design of the cantilever exerts a dramatic influence on its resonance response, the vibration mode must be analytically discussed further. (ii) The width effect induced by the plate structure cannot be disregarded because it exerts a significant influence on the resonance behavior of the cantilever. In this sense, Eq. S(5) displayed in Supplementary Information is sound for the two-dimensional modes where *W*(*y*) ≠ 1.

**Fig. 3:**
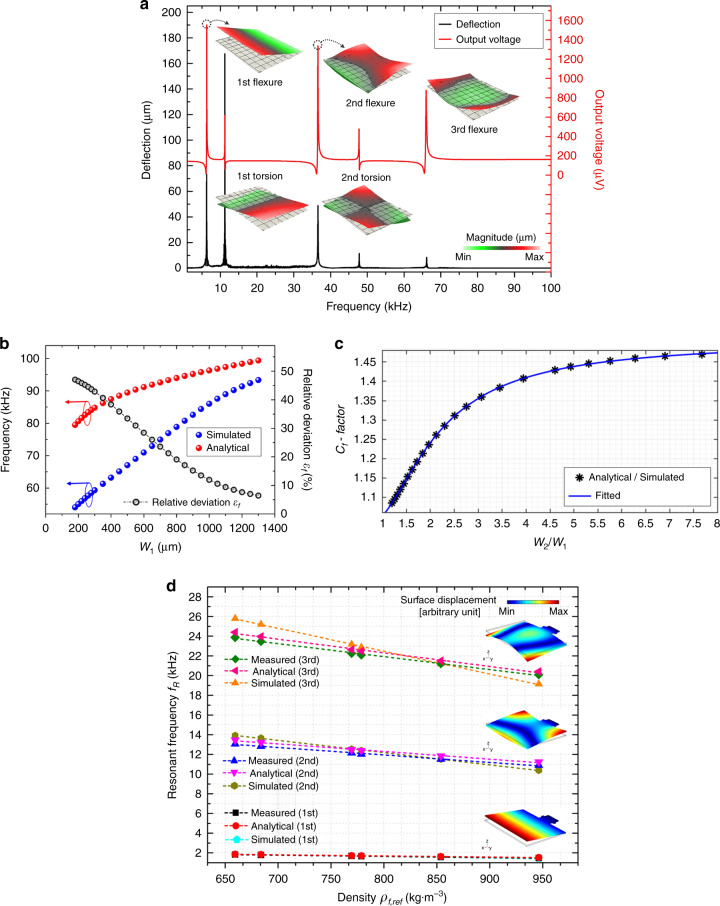
Modal verification of the out-of-plane modes of the cantilever. **a** The frequency responses consist of the deflection (left axis, lower curve) and output voltage (right axis, upper curve). **b** Difference between the analytical and simulated results of the resonant frequency under 2nd flexural mode. **c** The correction factor of the analytical resonant frequency as a function of the *W*_2_/*W*_1_. **d** Comparison of the resonant frequencies of stepped piezo-cantilever resonator under different vibration modes and in different liquids measured by analytical, simulated and experimental methods.

The frequency responses of the piezo-cantilever in terms of its deflection and output voltage are measured using a Polytec MSA500 scanning laser Doppler vibrometer and a SR830 lock-in amplifier, respectively. Figure 3a shows the independent vibration modes of the cantilever within five orders. To clarify, only the vibration of Region II is described because Region I primarily acts on stiffness, while Region II plays a crucial role in the mode shape of the stepped piezo-cantilever. The first flexure is the fundamental mode, which emits a slender beam-like vibration. Other higher-order vibrations exhibit the mode shape along the large width in varying extents, which verifies two-dimensional modes. The higher-order flexural and torsional modes have completely different appearances with respect to typical cantilevers. When comparing the electrical output characteristic, the voltage peak outputted by the flexural mode is larger than the torsional peak, making the flexure more attractive to achieve precise sensing in liquid media. To verify the modal analysis, the 2nd and 3rd flexural modes are of concern. Meanwhile, the 1st flexural mode is used for comparison.

#### Modal correction for the 2nd flexural mode

For the 2nd flexural mode of the cantilever, deformation clearly occurs along the width, particularly along with the stepped interface width at O_2_ Fig. 1a). The considerable difference between the widths of Regions I and II generates a stepped width effect acting on cantilever vibration. The correction factor is introduced to offset the deviations between the theoretical and experimental results. COMSOL Multiphysics software is used as the finite element method tool to illustrate the stepped width effect on cantilever vibration. The width (*W*_2_) of Region II is constant, while the width (*W*_1_) of Region I variably increases to taper the stepped width. Figure 3b depicts the analytical and simulated resonant frequencies along with their relative errors. The ratio of the resonant frequency between the theoretical and simulation results is shown in Fig. 3c. In the case of the relative errors below 10%, the analytical results are acceptable for the ratio *ζ*_W_ = *W*_2_/*W*_1_ reaching 1.25, indicating that the basic one-dimensional modeling can be reasonably approximated for the 2nd flexural mode. However, a higher *ζ*_*W*_ (*ζ*_*W*_ > 1.25) makes the stepped edge of Region II easier to generate additional vibration when the vibration of the cantilever has lower modal stiffness. Then, the frequency resonant under the 2nd flexural mode should be corrected by the factor *C*_*f*_, which depends on the ratio *ζ*_*W*_ *= W*_2_*/W*_1_. In addition, its expression is concluded with a fitting algorithm over the calculated values (black points in Fig. 3c):

1 $$C_f(\zeta _W) = \frac{{f_{\rm{anal}}}}{{f_{\rm{simu}}}} = \frac{{1.51\zeta _W^2 - 1.835\zeta _W + 2.149}}{{\zeta _W^2 - 1.089\zeta _W + 1.826}}.$$

#### Modal correction for the 3rd flexural mode

It is noted that the 3rd flexural mode of the stepped piezo-cantilever is a combination of vibrations that occur simultaneously along the length and width of the cantilever plate (region II). It can be divided into the 1st length flexure and 1st width flexure modes but without interdependence. To model this novel mode shape, the free–free length vibration based on flexural mode of the cantilever should be coupled in the overall shape function $\bar W_t$, which can be presented in the following^32–34^:

2 $$\begin{array}{l}\bar W_t(x,y) = \bar W_1(x)\bar W_1(y) = \bar W_1\\\left[ {(x){{{\mathrm{cos}}}}\kappa _1x + {{{\mathrm{ch}}}}\kappa _1x - 0.9819({{{\mathrm{sin}}}}\kappa _1x + {{{\mathrm{sh}}}}\kappa _1x)} \right],\end{array}$$

where the frequency parameter *κ*_1_ is 3*π*/(2*W*_2_). $\bar W_1(x)$ and $\bar W_1(y)$ represent the normalized displacements of Region II under the first-order vibrations in *x-* and *y-* directions, respectively. $\bar W_1(x)$ can be defined by above analytical results from Eqs. S(7) to (11) (in the Supplementary Information) and Table 1.

The numerical method based on energy principle can be utilized for predicting eigenmodes of the stepped piezo-cantilever with the two spatial coordinates. Region II is still dominant in the 3^rd^ flexural mode of the stepped piezo-cantilever, and its peak kinetic energy *T*max and peak strain energy *U*max can be expressed as:

3 $$T_{\max } = \rho _cT\omega _{11}^2{\int}_0^{L_2} {{\int}_0^{\frac{{W_2}}{2}} {\overline {W_t} ^2} } dxdy,$$

4 $$\begin{array}{l}U_{\max } = D{\int}_{0}^{L_2} {\int}_{0}^{\frac{{W_2}}{2}} \left\{ \left( {\nabla ^2\overline {W_t} } \right)^2 - 2\left( {1 - v_c} \right)\right.\\\qquad\quad\left.\left[ {\frac{{\partial ^4\overline {W_t} }}{{\partial x^2\partial y^2}} - \left( {\frac{{\partial ^2\overline {W_t} }}{{\partial x\partial y}}} \right)^2} \right] \right\} dxdy,\end{array}$$

where *ω*11 is the resonant frequency in the 3rd flexure mode of the stepped piezo-cantilever.

Additionally, the equation *T*_max_ = *U*_max_ is satisfied for the stepped piezo-cantilever at the resonance. The substitution of Eq. (2) into Eq. (3) and Eq. (4) brings about the optimal solution of the 3rd flexural resonant frequency.

### Modal verification

The first three-order flexural vibrations of the stepped piezo-cantilever are analyzed, accompanying the modeling correction for the width effect under the specific order. The corrected natural resonant frequencies of the stepped piezo-cantilever are listed as *f*_vac−1_ = 7.964 kHz, *f*_vac-2_ = 57.194 kHz, and *f*_vac-3_ = 103.603 kHz. In parallel, the experimental resonant frequencies of the stepped piezo-cantilever resonator are measured in six different liquids, the densities and dynamic viscosities of which are obtained from the Reference Fluid Properties (REFPROP) software, as shown in Table 2.

**Table 2:** Density and dynamic viscosity of different liquid media referenced by REFPROP at 20 °C

Medium	Density *ρ*_ref_	Dynamic viscosity *μ*_ref_
	(kg/m^3^)	(cP)
N-hexane	659.36	0.3123
N-heptane	683.82	0.4114
Methylcyclohexane	769.44	0.7288
Cyclohexane	778.68	0.9590
MD2M	853.74	1.4204
D4	946.31	2.5651

On the condition that the mode shape variation affected by liquid flow is sufficiently small to be neglected, the corresponding resonant frequency of the stepped piezo-cantilever resonator immersed in liquids can be derived, as shown in Fig. 3d. The resonant displacements of the stepped piezo-cantilever are simulated by COMSOL software and displayed by the inserted figures. Compared with the experimental results, the simulated results achieve a maximum absolute relative error of 4.61% for the 1st flexure, 6.56% for the 2nd flexure, and 8.02% for the 3rd flexure. For analytical results, the maximum values of the absolute relative error are 3.54% for the 1st flexure, 3.11% for the 2nd flexure, and 2.14% for the 3rd flexure. The analytical results exhibit excellent agreement with the experimental results, verifying the modal modeling for basic and two-dimensional flexural modes. The undamped frequencies are verified as well, which is feasible to simplify the estimated equation for the experimental densities in subsequent chapters.

## Results and discussion

### Performance characterization

**Fig. 4:**
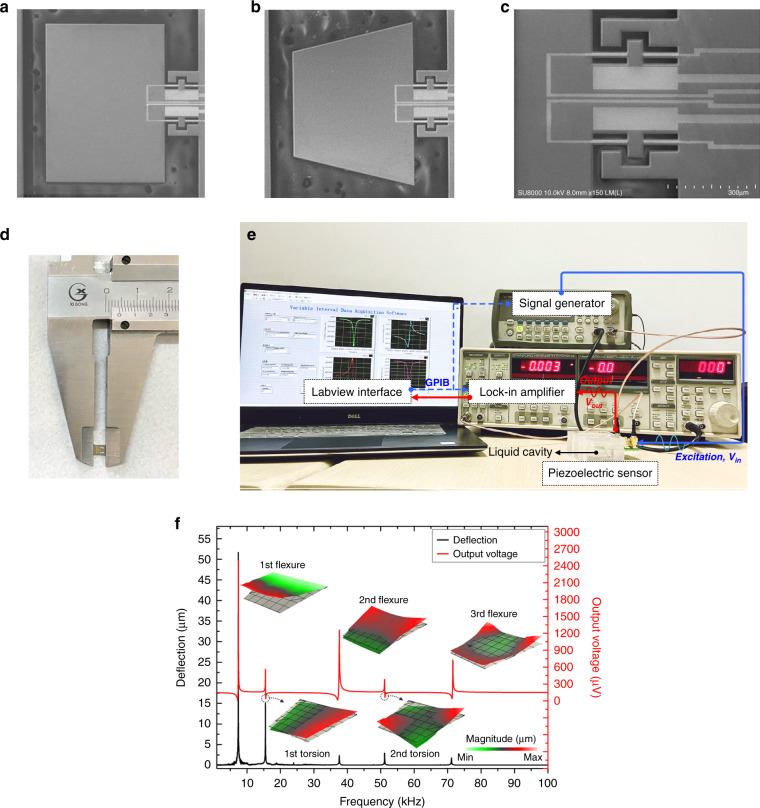
Fabricated sensing chip and experimental platform. **a**, **b** SEM image of the fabricated resonator with rectangular and trapezoidal stepped piezo-cantilevers, respectively. **c** SEM image of the actuation and sensing electrodes of the resonators. **d** The size of the fabricated sensing chip with the proposed resonator. **e** Illustration of the experimental setup for the stepped piezo-cantilever resonators. **f** Verification of the trapezoidal stepped piezo-cantilever resonator in the air under the out-of-plane modes, including the frequency responses of the deflection (left axis, lower curve) and output voltage (right axis, upper curve)

The mode shape as well as its resonant frequency of the cantilever resonator are key characteristics for liquid-phase sensing applications, such as the density and viscosity determination. The experiments with various liquid samples (listed in Table 2) are implemented to characterize the sensing behaviors of the stepped piezo-cantilever resonator. The stepped piezo-cantilever resonators with different shapes and vibration modes immersed in a small volume of liquid are investigated, including the stepped piezo-cantilever with a rectangular plate (as mentioned earlier) and trapezoidal plate, and their resonance at 2nd and 3rd flexural modes. The fabricated resonators are shown in Fig. 4a–c. Differentiating with the rectangular stepped piezo-cantilever, the trapezoidal stepped piezo-cantilever has a free side in the *x*-direction with a width of 900 μm. In Fig. 4d, the proposed sensing chip was fabricated with dimensions of only 3.4 mm × 4.2 mm, which is smaller than some reported density and viscosity chips^9,10,16^. This makes the sensing chip achieve an advantageous capacity in fluid monitoring, such as the embedded installation for miniaturized devices. In addition, the piezoelectrically induced voltage is used as the electric output of the chip but without the circuit compensation, which is available for chip-size reduction and enhancing the convenience of the sensor device.

The experimental setup is shown in Fig. 4e. The experimental temperature is controlled within approximately 20 °C ± 0.3 °C. The actuation electrodes of the stepped piezo-cantilever resonator are powered by a 33220A Agilent signal generator with an alternating excitation voltage of 2 V, and large voltage outputs can be obtained from the sensing electrodes. The stepped piezo-cantilever resonator chip is bonded on a PCB board and mounted into a customized polymethyl methacrylate shell with a 5 mL liquid cavity for immersion measurement.

The mode shape of the trapezoidal stepped piezo-cantilever resonator is also verified in air by a laser Doppler vibrometer and compared with the piezoelectric output, as shown in Fig. 4f. If the resonator under the 2nd and 3rd flexural modes has more outstanding amplitudes, it is more similar to the rectangular stepped piezo-cantilever.

**Fig. 5:**
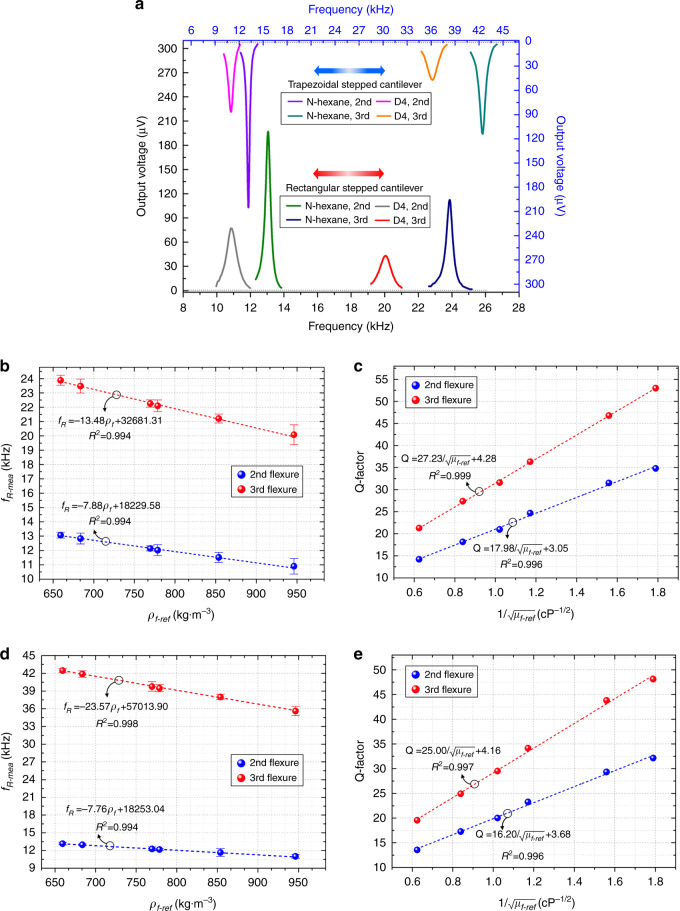
Sensing characteristics of rectangular and trapezoidal stepped piezo-cantilever resonators. **a** Frequency responses of different resonators under the 2nd and 3rd flexural modes in n-hexane and D4 medium. **b**, **c** The measured resonant frequency and Q-factor of the rectangular stepped piezo-cantilever resonator are the function of the liquid density and viscosity, separately. **d**, **e** The measured resonant frequency and Q-factor of the trapezoidal stepped piezo-cantilever resonator are the function of the liquid density and viscosity, separately. The error bars represent the standard deviation of the averaged frequencies.

The frequency responses of the rectangular and trapezoidal stepped piezo-cantilever resonators in liquids are shown in Fig. 5a. The n-hexane and D4 medium are selected to validate the maximum change in output amplitude. Notably, the resonance peak of the cantilevers at higher orders is sufficiently distinct to be detected precisely. This condition can be attributed to the small parasitic component and leakage current. Thus, complex electronic circuits will have no requirement for signal compensation.

Figure 5b–e show the sensing characteristics of different stepped piezo-cantilever resonators under the 2nd and 3rd flexural modes in liquids. The attractive linearities occur on both between the density and resonant frequency, and between the viscosity and Q-factor of the stepped piezo-cantilever. In addition, their linear correlation coefficients exceed 0.994. The liquid density contributes to the variation of the resonant frequency, while the viscosity (1/√μ) is the dominance in Q-factor reduction rather than the viscosity-density product (1/√ρμ) nonlinear functions. These critical behaviors enable the proposed resonator chip to determine the two liquid quantities separately. The variation slope of the resonant frequency with the density can be employed to estimate the mass change sensitivity (MCS). The MCS represents the minimum liquid mass change that can be discernable under a resonant frequency resolution, which can be expressed as:

5 $${{{\mathrm{MCS}}}} = V_f{\frac{\Delta \rho _f}{\Delta f_R}}$$

where *V*_*f*_ is the liquid occupying volume that surrounds the stepped piezo-cantilever. This can be concretized as a circular cylinder^35,36^ with a diameter and height equal to the dominant scale along the width and length directions of the stepped piezo-cantilever. The rectangular stepped piezo-cantilever resonator responds to an MCS of 268 ng/Hz and 156 ng/Hz under the 2nd and 3rd flexural vibration modes, respectively. The trapezoidal stepped piezo-cantilever resonator responds to an MCS of 272 ng/Hz and 91 ng/Hz under the 2nd and 3rd flexural vibration modes, respectively. Additionally, the frequency instability is evaluated by the standard deviations. For the rectangular cantilever resonator, the maximum frequency instability is 0.05‰ at the 2nd flexure and 0.03‰ at the 3rd flexure. For the trapezoidal cantilever resonator, the calculated values are 0.05‰ at the 2nd flexure and 0.02‰ at the 3rd flexure. These results indicate that the stepped piezo-cantilever resonator is a fine potential choice for (bio) chemical liquid sensing.

Considering that the Q-factor can be linearized by the viscosity transformation of the liquid, the estimation of the measured viscosity *μ*_*f,e*_ can be expressed as:

6 $$\mu _{f,e} = \frac{{\chi _1}}{{f_R^3\rho _{f,e}}}\left( {\frac{1}{{Q_L}} - \frac{1}{{Q_A}}} \right)^2$$

where *ρf,e* denotes the measured density, *χ*1 is the calibration coefficient that can be determined by experimental and reference values, and *QL* and *QA* are the Q-factors of the stepped piezo-cantilever resonator in liquid and air immersion, respectively. The *QA* can be derived in Figs. 2a and 3f, and its values for the rectangular stepped piezo-cantilever resonator are 419 (2nd flexure) and 601 (3rd flexure); for the trapezoidal stepped piezo-cantilever resonator, they are 526 (2nd flexure) and 825 (3rd flexure). Based on the well-known inviscid theory, the relationship of fluid density and resonant frequency of the slender cantilever (whose length/width greatly exceeds unity) in vacuum and fluid immersions is presented as^17^:

7 $$\rho _f = \frac{{4\rho _cT}}{{\pi W_d}}\left( {\frac{{f_{\rm{vac}}^2}}{{f_R^2}} - 1} \right).$$

The empirical corrections should be developed for the cantilever that is affected by the finite aspect ratio (length/width) with respect to the classic fluid–structure interaction. The resonant frequency of the cantilever in vacuum as well as corresponding eigenmodes have been verified early, whose width effect coupled with different vibrations have been optimized. Thus, we induce the calibration factor *χ*^2^ to substitute for the other parameters, which are related to the specific cantilever material and dimensions. Thus, the estimation equation of *ρ*_*f,e*_ can be presented as:

8 $$\rho _{f,e} = \chi _2\left( {\frac{{S_c^2f_{\rm{vac}}^2}}{{f_R^2}} - 1} \right).$$

Only one calibration liquid can provide the two calibration coefficients *χ*_1_ and *χ*_2_ in this study to simplify the density and viscosity estimations for the rectangular stepped piezo-cantilever resonator. The cross-section factor *S*_*c*_ in Eq. (8) is feasible for the cantilever plates with variable widths, such as the trapezoidal and triangular structure. In addition, the *S*_*c*_ can be employed as a constant that depends on specific geometries^37^ and vibrational modes of the trapezoidal stepped piezo-cantilever resonator. The values of *f*_vac_ can be equal to the rectangular stepped piezo-cantilever, and another calibration liquid is used for *S*_*c*_ determination. The calibration parameters are given in Table 3. The measurement values of density and viscosity by different resonators, along with their deviations between them and reference values, are presented in Tables 4 and 5.

The rectangular and trapezoidal stepped piezo-cantilever resonators achieve attractive measurement accuracies for different liquid quantities. The density average deviations of the rectangular stepped piezo-cantilever resonator are 0.72% and 0.39% under the 2nd and 3^rd^ flexural modes, respectively. In addition, the viscosity average deviations are 6.19% and 4.95% under the two order modes, respectively. In parallel, the density average deviations of the trapezoidal stepped piezo-cantilever resonator are 0.63% and 0.45% under the 2nd and 3rd flexural modes, respectively. In addition, the average deviations for the viscosity are 4.74% and 3.50% under the two order modes, respectively. The higher-order flexural vibration obtains better measurement performance for the same-shape cantilever. The decoupled measurement equations for the density and viscosity are also important factors for enhancing the sensing precision, which confirms the advantages of the proposed stepped piezo-cantilever resonators.

**Table 3:** Values of the calibration coefficients for different stepped piezo-cantilever resonators

	*χ*_1_(cP·Hz^3^ kg m^−3^ × 10^17^)	*χ*_2_(Hz^2^ kg m^-3^)	*S*_*c*_
Rectangular, 2nd	6.331	35.997	/
Rectangular, 3rd	89.641	37.106	/
Trapezoidal, 2nd	5.815	12.850	1.669
Trapezoidal, 3rd	430.539	78.723	1.258

**Table 4:** Density *ρ*_*f,e*_ and viscosity *μ*_*f,e*_ values estimated by the rectangular stepped piezo-cantilever resonator, where the deviations *ε*_*ρ*_ and *ε*_*μ*_ denote the absolute relative errors between the experimental and reference values.

Medium	Mode	^*ρ*^*f,e* (kg m^−3^)	^*μ*^*f,e* (cP)	^*ε*^*ρ* (%)	^*ε*^*μ* (%)
N-hexane	2nd	653.78	0.3014	0.85	3.50
	3rd	661.41	0.2947	0.31	5.63
N –heptane	2nd	679.13	0.3797	0.69	7.69
	3rd	685.41	0.3928	0.23	4.49
Methylcyclohexane	2nd	762.58	0.6747	0.89	7.39
	3rd	767.19	0.7098	0.29	2.61
Cyclohexane^a,b^	2nd	778.68	0.9590	0	0
	3rd	778.68	0.9590	0	0
MD2M	2nd	852.03	1.3514	0.20	4.86
	3rd	848.27	1.3467	0.64	5.19
D4	2nd	955.37	2.3717	0.96	7.54
	3rd	950.79	2.3904	0.47	6.81

^a,b^Indicates the referenced viscosity and density for calibration procedure, respectively.

**Table 5:** Density *ρ*_*f,e*_ and viscosity *μ*_*f,e*_ values estimated by the trapezoidal stepped piezo-cantilever resonator, where the deviations *ε*_*ρ*_ and *ε*_*μ*_ denote the absolute relative errors between the experimental and reference values.

Medium	Mode	*ρ*_*f,e*_ (kg m^−3^)	*μ*_*f,e*_ (cP)	*ε*_*ρ*_ (%)	*ε*_*μ*_ (%)
N-hexane	2nd	663.27	0.3279	0.59	4.98
	3rd	662.95	0.3249	0.54	4.03
N -heptane^b^	2nd	683.82	0.4043	0	1.71
	3rd	683.82	0.4006	0	2.61
Methylcyclohexane	2nd	765.45	0.6945	0.52	4.71
	3rd	766.82	0.7039	0.34	3.41
Cyclohexane^a,b^	2nd	778.68	0.9590	0	0
	3rd	778.68	0.9590	0	0
MD2M^b^	2nd	849.04	1.3554	0.55	4.58
	3rd	850.26	1.4051	0.41	1.08
D4	2nd	954.60	2.3675	0.88	7.70
	3rd	941.56	2.4021	0.50	6.36

^a,b^Indicates the referenced viscosity and density for calibration procedure, respectively.

The performance comparison of this work and other reported resonator-based density and viscosity sensors^38,39^ is shown in Table 1S of Supplementary Information. Compared with other studies, the proposed sensor with piezoelectric self-actuation and self-sensing capabilities has achieved comprehensively high performances in measurement accuracy, density sensitivity, and viscosity within the allowable range.

The accuracy of the resonant device has a significant dependence on the resonator design, while a higher Q-factor allows for wider viscosity determination. The Q-factor of our sensing device can be improved by adopting a more complex fluid–structure interaction model, not only to mitigate fluid damping via dimensional alteration but also to reduce its decay rate, which is related to liquid viscosity.

### Optimization consideration

**Fig. 6:**
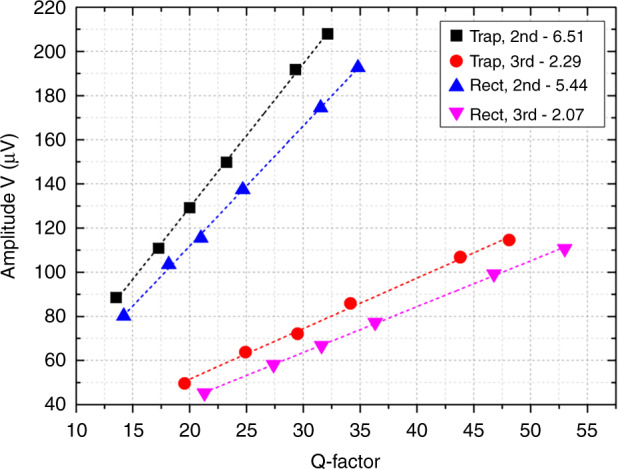
Characteristics of the output voltage amplitude for different stepped piezo-cantilever resonators. The dashed line represents the linear fit, and their slopes *ΔV*/*ΔQ* are indicated in the legend. The *ΔV*/*ΔQ* is defined as the VQR parameter of the resonator to represent the decay rate of the Q-factor.

The characteristics of the output amplitudes for the stepped piezo-cantilever resonators at the resonance are summarized in Fig. 6. The changes ratio of amplitude *V* to the Q-factor (VQR=*ΔV/ΔQ*) in liquids is a constant for a given stepped piezo-cantilever shape and resonance mode, which can be defined as the decay rate of the Q-factor of resonators. The stepped piezo-cantilever resonator with a lower VQR value is able to sense higher viscous liquid media if the Q-factor can be acceptably measured by electrical characterization. On the other hand, the VQR also requires a balance between the voltage output and the resonance peak. A higher-order mode generally leads to a weaker resonance output, though a high Q-factor can be maintained. The VQR consideration provides several optimization points to the piezoelectric resonator under higher-order vibrations. First, the parasitic effects, which mainly result from the parallel capacitance and serial resistance, should be reduced, and the piezoelectric strain coefficient *d*_31_ should be increased. In this study, the trapezoidal stepped piezo-cantilever resonator under 3rd flexural mode has a lower Q-factor than the rectangle stepped piezo-cantilever resonator, whereas it has approximately 1.8 times higher resonant frequency than the rectangle stepped piezo-cantilever resonator. This result is possibly due to the unequal material parameters for the stepped piezo-cantilevers, especially the AlN piezoelectric layer. The doped AlN deposition^40^ has been proven to increase the piezoelectric coefficient. In addition, a higher-order resonance is still preferred, and the actuated and sensing electrodes can be further optimized to fit the specific mode shape to realize high-precision operation for a microresonator immersed in viscous liquid media.

## Conclusion

A stepped piezo-cantilever resonator with AlN material is presented to excite two-dimensional flexural modes for liquid quantity determination. The model is based on an analysis of the flexural vibrations of the resonator, and it consists of a modeling correction for the width effect acting on the resonance. The resonant frequencies of the resonator under different flexural modes are predicted in vacuum and liquid immersion. The relative errors for the resonant frequency between the model prediction and experimental results are less than 4%. The experiment indicates that the density and viscosity of the liquid can be separately linearized by the sensing characteristic of the resonator, which allows for decoupled determination based on theoretical analyses. The experimental results indicate accuracies of 0.39% for density and 3.50% for viscosity in working ranges of 659.36–946.31 kg m^−3^ and 0.31–2.57 cP, respectively. In view of the output characteristic, the VQR is proposed for the piezo-cantilever resonator to pursue well-balanced electrical access at higher-order resonances. These results provide optimization guidance for the efficient design of self-actuated and self-sensing piezoelectric resonators, making them a powerful alternative in liquid monitoring.

## Supplementary information

Piezoelectric-AlN resonators at two-dimensional flexural modes for density and viscosity decoupled determination of liquids
