# Supplementary material for: Piezoelectric-AlN resonators at two-dimensional flexural modes for the density and viscosity decoupled determination of liquids
Source: Microsyst Nanoeng. 2022 Apr 2;8:38. doi: 10.1038/s41378-022-00368-0 (PMC8976841; doi:10.1038/s41378-022-00368-0)
Supplement: Supplementary file 1 — Piezoelectric-AlN resonators at two-dimensional flexural modes for density and viscosity decoupled determination of liquids [file 41378_2022_368_MOESM1_ESM.pdf]

Piezoelectric-AlN resonators at two-dimensional flexural modes for density and viscosity decoupled determination of liquids

SUPPLEMENTARY MATERIAL

Theoretical analyses

The governing equation for the dynamic deflection $w= w\,(x, y, t)$ in z-direction is[29]

$$D\nabla^2\nabla^2 w + \rho_c T \frac{\partial^2 w}{\partial t^2} = 0, \tag{1}$$

where ρ_c is the mass density of cantilever material and D is the flexural rigidity of the cantilever, and D can be presented as:

$$D = \frac{ET^3}{12(1 - {v_c}^2)}, \tag{2}$$

where E is the Young's modulus and v_c is the Poisson's ratio of the cantilever material.

Under the simple harmonic excitation, the solution w can be expressed in form of $w(x, y, t)=W(x, y)e^{j\omega t}$, in which ω is the undamped natural frequency, $W(x, y)$ is the shape function of the cantilever in a certain vibration mode. Then Eq. (1) can be transformed into following expression:

$$\nabla^2\nabla^2 W - \beta^4 W = 0, \tag{3}$$

where β is a function of the resonant frequency of the cantilever, presented as follows:

$$\beta^4 = \frac{\rho_c T}{D}\omega^2. \tag{4}$$

The cantilever is composed of a plate structure, thus, the $W(x, y)$ can be well approximated in a separable variable form[31]:

$$W_{ij}(x, y) = W_i(x) W_j(y), \quad i, j \in N \tag{5}$$

where i and j denote the number of flexural half-waves along x- and y- directions of the cantilever, respectively. And $W_i(x)$ and $W_j(y)$ are the independent shape functions at one dimension. The general solution for Eq. (5) can be presented as follows[29]:

$$\begin{cases} W_i(x) = A_m \cos\lambda_i x + B_m \mathrm{ch}\lambda_i x + C_m \sin\lambda_i x + D_m \mathrm{sh}\lambda_i x, i \in N^* \\ W_j(y) = 1, j = 0 \\ W_j(y) = A_m \cos\kappa_j y + B_m \mathrm{ch}\kappa_j y + C_m \sin\kappa_j y + D_m \mathrm{sh}\kappa_j y, j \in N^*, \end{cases} \tag{6}$$

where coefficients A_m, B_m, C_m, and D_m are constants determined by the boundary conditions. λ_i and κ_j are the natural frequency parameters of a certain mode shape. In particular, given that the two opposite lengths of the cantilever are free, the case of j=0 represents that the fundamental mode of the plate-structural cantilever can be approximated by one-dimensional model. Then the gap of the resonance variation induced by width can be disregarded or corrected by empirical approaches[32,33]. An efficient method for simplifying Eqs. (5) and (6) and obtaining natural frequency more conveniently is developed. Thus, further analytical investigation is performed for transverse motions to focus on the deformation along the length of the cantilever. Based on Eq. (6), the displacements along x-direction of the two distinct regions I and II in the cantilever are defined as follows:

$$\begin{cases} W_I(x) = A_1 \cos\beta_i x + B_1 \mathrm{ch}\beta_i x + C_1 \sin\beta_i x + D_1 \mathrm{sh}\beta_i x, x \in [0, L_1], \\ W_{II}(x) = A_2 \cos\beta_i x + B_2 \mathrm{ch}\beta_i x + C_2 \sin\beta_i x + D_2 \mathrm{sh}\beta_i x, x \in [0, L_2], \end{cases} i \in N^* \tag{7}$$

where β_i represents a certain β function when the cantilever vibrates under the ith resonance mode. To satisfy the conditions of continuity at O_2, the transverse deflections, bending moments, the first derivatives of the deflection, and transverse shear forces at the interface between the two regions must follow:

$$\begin{cases} W_{\mathrm{I}}(L_1) = W_{\mathrm{II}}(0), \\ M_{x\mathrm{I}}(L_1) = M_{x\mathrm{II}}(0), \\ \dfrac{dW_{\mathrm{I}}}{dx}\Big|_{x=L_1} = \dfrac{dW_{\mathrm{II}}}{dx}\Big|_{x=0}, \\ Q_{x\mathrm{I}}(L_1) = Q_{x\mathrm{II}}(0), \end{cases} \tag{8}$$

where the bending moments M_x and the transverse shear forces Q_x are defined as follows:

$$M_x = -EI_m \frac{\partial^2 W}{\partial x^2}, \tag{9}$$

$$Q_x = \frac{\partial M_x}{\partial x} = -EI_m \frac{\partial^3 W}{\partial x^3}, \tag{10}$$

where I_m is the area moment of inertia of the regional cross-section.

For the cantilever with clamped-free (CF) boundary, the regions I and II are further restrained by following boundary conditions,

$$\begin{cases} W_{\mathrm{I}}(0) = 0, \\ \dfrac{dW_{\mathrm{I}}}{dx}\Big|_{x=0} = 0, \\ M_{x\mathrm{II}}(L_2) = 0, \\ Q_{x\mathrm{II}}(L_2) = 0. \end{cases} \tag{11}$$

To refine the mode function of Region II, the eight parameters A_m, B_m, C_m, D_m, (m = 1, 2) can be reduced into two coefficients, and the other parameters are expressed by these two. That is, the continuity conditions between Regions I and II outlined in Eq. (8) provide the four required equations, while the clamped support conditions outlined in Eq. (11) provide the other two. The free boundary related to Region II in Eq. (11) forms a singular coefficient matrix. The solution of the matrix is the eigenvalue β with infinite number of roots, which can be searched by iterative procedures based on linear interpolation. Then the corresponding nature frequencies f_{vac} under out-of-plane flexural modes as well as the rough mode shape of the cantilever in vacuum can be derived by Eqs. (4) and (7).

In accordance with the fluid-structure interaction theory, the cantilever resonance immersed in a viscous liquid medium can be derived by its natural frequency[18] as follows:

$$f_R = \frac{\sqrt{8\pi^2 f_{vac}\left(1+Lg_2/M_c\right)-\left(1+Lg_1/M_c\right)^2}}{2\sqrt{2}\pi\left(1+Lg_2/M_c\right)} \tag{12}$$

where f_R is the resonant frequency of the cantilever immersed in liquid. M_c is the cantilever mass, g_1 is the liquid damping coefficient, and g_2 is the displaced mass of the liquid per unit length of the cantilever in liquid immersion. This expression is defined under the condition that viscous damping is the dominant damping of the cantilever. And g_1 and g_2 can be expressed by the Reynolds number (Re) of the liquid flow surrounding the cantilever and the hydrodynamic function, as follows:

$$g_1 = \pi\mu_f Re\Gamma_I(Re), \tag{13}$$

$$g_2 = \frac{\mu_f Re\Gamma_R(Re)}{2f_R}, \tag{14}$$

where $Re = \pi\rho_f W_d^2 f_R/(2\mu_f)$, where W_d is the dominant width scale in the hydrodynamic flow, ρ_f and μ_f are the liquid density and viscosity, respectively. Γ_I and Γ_R are the real and imaginary components of the hydrodynamic function:

$$\Gamma(\omega) = \Omega(\omega)\left[1+\frac{4jK_1\left(-j\sqrt{jRe}\right)}{\sqrt{jRe}K_0\left(-j\sqrt{jRe}\right)}\right] \tag{15}$$

where K_0 and K_1 are the zero-order and one-order modified Bessel functions of the second kind, respectively, and $\Omega(\omega)$ is the correction function associated with the cross section of the cantilever. The $\Omega(\omega)$ is close to 1 owing to the Re~10^7 of the proposed cantilever. Therefore, based on the undamped resonant frequency f_{vac} is substituted into Eqs. (12) to (15), the resonant frequency f_R of the cantilever immersed in liquid can be derived from an implicit equation.

Table 6 Performance comparison of various density and viscosity sensors

	Density sensitivity	Maximal density deviation	Viscosity range	Maximal viscosity deviation	Actuation/sensing principle
Wilson et al.[38]	50 $pg\cdot Hz^{-1}$	3.5%	1-2.4 cP	10%	Piezoelectric actuation/ sensing
Khan et al.[7]	16 Hz $kg^{-1}\cdot m^3$	/	0.9-2.4 cP	0.45%	Piezo-actuator/ optical deflection
Toledo et al.[26]	/[a]	0.7%	1.71-1.97 cP	16.7%	Piezoelectric/ admittance
Oliva et al.[39]	/[a]	1.5%	1-219 cP	9.6%	Photo-thermal/ optical deflection
This work	91 $ng\cdot Hz^{-1}$/ 24 Hz $kg^{-1}\cdot m^3$	0.96%	0.31-2.57 cP	7.7%	Piezoelectric actuation/ sensing

[a] Indicates that the resonant frequency has nonlinearity with the density, and the density sensitivity cannot be precisely evaluated.
